# Quality of Trauma Surgery Podcasts in Credibility, Content, and Design

**DOI:** 10.1001/jamanetworkopen.2024.15636

**Published:** 2024-06-20

**Authors:** Asma Altaf Hussain Merchant, Shayan Ali Shah, Asfia Arham Khursheed, Madeeha Ali, Sohaib Najam, Rimsha Farooq, Saqib Kamran Bakhshi, Noreen Afzal, Komal Abdul Rahim, Namra Qadeer Shaikh, Adil H. Haider

**Affiliations:** 1 Dean’s Office, Medical College, Aga Khan University, Karachi, Pakistan; 2 Department of Surgery, Aga Khan University, Karachi, Pakistan; 3 Department of Community Health Sciences, Aga Khan University, Karachi, Pakistan

## Abstract

**Importance:**

There has been an emerging trend of podcast use in medical education over the past decade. With the increasing number of podcasts and learners’ interest in trauma surgery, it is vital to validate the quality of these podcasts.

**Objective:**

To determine the quantity and quality of podcast episodes on trauma surgery.

**Design, Setting, and Participants:**

This cross-sectional study identified trauma surgery podcasts published globally on the Google, Apple, and Spotify platforms as of May 31, 2023. An initial screening excluded podcasts not fulfilling the inclusion criteria (published as of May 31, 2023, and covering clinical knowledge or mentorship on general trauma surgery). A 10-item checklist developed from a modified Delphi consensus method was created to assess the podcasts’ credibility, content, and design as indicators of quality. Data extracted from each podcast episode included the duration, setting, host and guest credentials, and fulfillment of quality indicators. Data were analyzed on August 13, 2023.

**Main Outcomes and Measures:**

The quality of each podcast was assessed using the 10-question checklist. A score of at least 80% in credibility (4 of 5 points), 75% in content (3 of 4 points), and 100% in design (1 of 1 point) was required to classify a podcast episode as having good quality.

**Results:**

The initial search identified 204 podcasts on trauma surgery, of which 55 podcasts met the inclusion criteria. All 55 podcasts were published after 2014, with the majority hosted by males (85% [61 of 72 hosts]), based in the US (91% [50 of 55 podcasts]), and focused on clinical knowledge (80% [44 of 55 podcasts]). Podcast hosts and guests predominantly held a Doctor of Medicine degree (83% [113 of 136]), with 72% [46 of 64] of guests identifying as practicing trauma surgeons. While all 55 podcasts had excellent quality in terms of content and design, 20% (11 of 55) of podcasts were rated poorly on credibility. Credibility was further reduced for 60% (33 of 55) of podcasts because of nondisclosure of conflicts of interest.

**Conclusions and Relevance:**

In this cross-sectional study of trauma surgery podcasts, most had good-quality content and design, but there was a lack of explicit conflict of interest disclosure. Content producers need to disclose their conflicts of interest appropriately to ensure credibility and improve the quality of their podcasts.

## Key Points

**Question:**

Do podcast episodes on trauma surgery have adequate quality for use in medical education?

**Findings:**

In this cross-sectional study of 55 trauma surgery podcast episodes, all episodes had excellent quality in terms of content and design, but 20% were rated poorly on credibility. Conflicts of interest were not disclosed in 60% of podcasts, further decreasing their credibility.

**Meaning:**

Findings of this study suggest that there is a need for content producers to clearly indicate their conflicts of interest to ensure credibility and improve the quality of their podcasts.

## Teaser

This cross-sectional study examines the quality of trauma surgery podcasts for use in medical education.

## Introduction

Over the past 10 years, podcast use has grown exponentially in medical education, especially during and after the COVID-19 pandemic. In a study of over 200 emergency medicine residents, 91% of respondents used podcasts, placing them among the most commonly used sources of online learning.^1^ Similarly, the number of podcasts increased to meet the rising number of learners. For example, in orthopedics, the quantity of podcasts increased 12-fold from 2016 to 2020.^2^ Support from numerous professional societies, such as the American College of Physicians, has made podcasts readily available to health care professionals,^3^ specifically residents, who consider podcasts to be a feasible and low-cost tool for learning.^4,5^ Such podcasts can further aid in disseminating information spanning a variety of scopes, such as clinical knowledge, practice management, and field-specific research.^2,6^

While the use of podcasts in medical education has continued to increase over the years, educators have recently started to emphasize the need to assess podcast quality through standardized processes. Several checklists have been developed to standardize secondary medical education resources, such as DISCERN and the Health on the Net Foundation Code of Conduct, which are quality scores for health care websites, and Preferred Reporting Items for Systematic Reviews and Meta-Analyses for systematic reviews.^7,8,9^ Similarly, Approved Instructional Resources(AIR) and Medical Education Translational Resources: Impact and Quality (METRIQ) were developed to assess Free Open Access Medical Education resources and blog posts, respectively.^10,11^ However, these checklists showed lower reliability than gestalt ratings,^11^ requiring the creation of revised AIR (rAIR) and revised METRIQ (rMETRIQ) tools, which increased their usability.^12,13^ Therefore, the use of a standardized quality assessment checklist is essential for evaluating educational podcasts as well. A systematic review of the literature revealed 151 indicators applicable to blogs and podcasts for assessing their quality.^14^ A modified Delphi consensus of 44 international health professions educators short-listed 10 specific indicators from this list that could ascertain the quality of podcasts in medical education.^15^ While this standardization could aid learners, educators, content producers, and academic leaders in ensuring the production and use of high-quality resources, the established criteria has not been used to ascertain the quality of medical education podcasts.

With the evolution of electronic resources in medical education, several podcasts have begun to focus on specialized surgical areas, including trauma surgery. It is vital to assess the quality of the increasing number of podcasts in trauma surgery before they are used by learners, mainly medical students and residents, who have a growing interest in this subspecialty. Therefore, this study was conducted to determine the characteristics of trauma surgery podcast episodes published to date and assess their quality.

## Methods

### Study Design and Data Collection

We conducted a cross-sectional study at the Aga Khan University Hospital, one of the largest academic medical centers in Pakistan, from June to August 2023. This study received an exemption from the Aga Khan University Hospital Ethics Review Committee because the collected data did not include patient information and were available for public use. We followed the Strengthening the Reporting of Observational Studies in Epidemiology (STROBE) reporting guideline.^16^ Data were collected from 3 podcast platforms: Google, Apple, and Spotify. The term *trauma surgery* was searched in each platform and duplicates were excluded. While these podcast platforms are available to the general public, the search term short-listed podcast episodes that are intended only for medical professionals and trainees as a source of information or mentorship.

All podcast episodes identified by this search underwent an initial screening for eligibility based on their title and summary. This preliminary check was performed by 2 of us (A.A.H.M. and S.A.S.). All podcast episodes published as of May 31, 2023, that focused on overall trauma surgery rather than specific subspecialties, and covered general or clinical knowledge and educational or mentorship aspects were included for review. Podcast episodes published as a conference keynote, those that focused on general surgery overall with only mention of a trauma surgery aspect, those that discussed specific subspecialties of trauma surgery (eg, orthopedic or neurological trauma), and those that covered psychological trauma, posttraumatic stress disorder, or posttrauma rehabilitation were excluded.

In-depth scrutiny of and data collection from the screened podcasts were conducted by surgical residents (A.A.K, M.A., S.N., and R.F.) who had baseline knowledge and prior exposure or experience in trauma surgery. Each podcast episode was evaluated by 2 surgical residents independently to ensure the reliability of the collected data. Prior to data collection, these residents underwent training to grasp the full scope encompassed by each indicator. This training helped reduce any potential bias and introduce uniformity in decision-making.

The extracted data included details of the podcast episode (date of publication, country where the podcast was published, scope of the podcast [eg, clinical knowledge, education or mentorship, or general]), details of the podcast hosts (credentials, designation of the host [eg, trauma surgeon, surgical resident]), details of the podcast guests where applicable (including credentials and designation), and quality indicators in 3 domains (credibility, content, and design). These quality indicators were extracted from a 10-question checklist derived after a modified Delphi consensus with health profession educators.^15^ The credibility domain included 5 questions focusing on transparency and trustworthiness of each podcast episode by inquiring about disclosures of the identity and conflicts of interest of the hosts and guests.^14,15^ The content domain focused on subject matter through 4 questions investigating the academic rigor and professionalism of the podcast episode. Lastly, the design domain had 1 question that aimed to explore the presentation and functionality of the podcast through resources used for its production.^14,15^ Data were extracted using REDCap (Vanderbilt University), and any podcast episode that did not fulfill the inclusion criteria was excluded.

When opposing answers were provided by the residents for the same podcast episode, a senior member of the research team (S.K.B.) resolved differences after thoroughly reviewing the episode. To ensure the quality of data collection, a senior member of the research team (S.K.B.) also performed random checks on 5% of included podcast episodes.

For quality indicators, we a priori hypothesized that each podcast episode would fulfill at least 75% of the indicators for each of the 3 domains of credibility, content, and design. Each indicator was scored as either *yes* (1 point) or *no* (0 points). Therefore, podcast episodes with a score of at least 80% in credibility (4 of 5 points), 75% in content (3 of 4 points), and 100% in design (1 of 1 point) were deemed as having good quality.

### Statistical Analysis

Data were analyzed using SPSS, version 21 (IBM Corp). Categorical data were reported as frequencies and percentages. Since the responses to quality indicators were binary (yes or no), frequencies (percentages) with 95% CIs were assessed for each question on the checklist. For the indicators with 100% *yes* responses, 1-sided 95% CI was reported. Data were analyzed on August 13, 2023.

## Results

**Figure 1.**
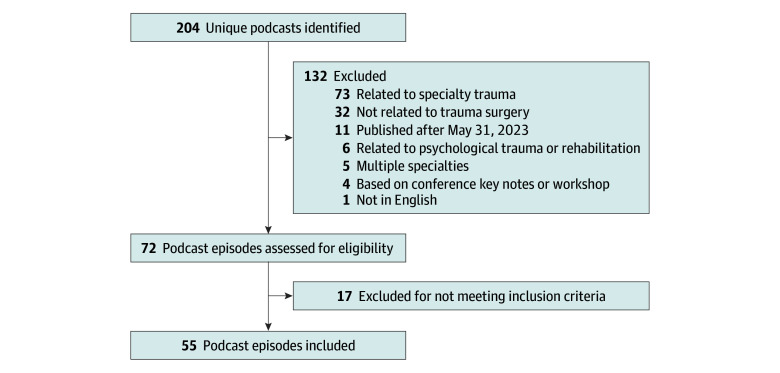
Study Flow Diagram

After removal of duplicates, a total of 204 unique podcasts relating to trauma surgery that met the eligibility criteria were identified. The initial screening revealed 72 eligible podcast episodes. Surgical residents identified 55 podcast episodes that met the inclusion criteria (Figure 1; eTable in Supplement 1). The senior surgical faculty member (S.K.B.) performed a quality check and resolved discrepancies. This quality check revealed a discrepancy rate of 4.2% within the responses of the residents for a given podcast episode, which indicated that the collected dataset had a reliability of 95.8%.

Table 1 shows the demographic characteristics of included podcast episodes, all of which were published after 2014. The mean (SD) duration of the episodes was 41.6 (22.1) minutes. Of the included 55 podcast episodes, most were from the US (n = 50 [91%]) and focused on clinical knowledge (n = 44 [80%]). The majority of the podcasts (n = 38 [69%]) had 1 host, while 17 episodes (31%) had 2 hosts, resulting in a total of 72 hosts for the 55 included podcast episodes. Among the 72 hosts, there were considerably more male hosts (61 [85%]) than female hosts (11 [15%]). A substantial portion of the hosts held a Doctor of Medicine (MD) degree (52 [72%]), with one-fourth of hosts being trauma surgeons (18 [25%]). While 12 of the 55 episodes (22%) did not feature a guest, those that did had a majority of guests with MD credentials (61 of 64 guests [95%]). A total of 46 of 64 guests (72%) were practicing trauma surgeons.

**Table 1.** Characteristics of Included Trauma Surgery Podcast Episodes

Characteristic	No. (%) (N = 55)
**Podcast (N = 55)**	
Podcast country of origin	
US	50 (91)
Canada	4 (7)
South Africa	1 (2)
Scope of podcast	
Clinical knowledge	29 (53)
Education or mentorship	10 (18)
Both clinical knowledge and education or mentorship	15 (27)
General	1 (2)
Subdomains of clinical knowledge^a^	44 (80)
Theoretical	30 (68)
Practical	39 (89)
Evidence-based	17 (39)
Subdomains of mentorship or education	25 (46)
Mentorship	18 (72)
Education	7 (28)
No. of podcast hosts	
1	38 (69)
2	17 (31)
No. of guests	
None	12 (22)
1	31 (56)
2	9 (16)
>2	3 (6)
**Hosts (n = 72)**	
Sex of podcast hosts	
Male	61 (85)
Female	11 (15)
Credentials of podcast hosts^a^	
MD	52 (72)
MPH	2 (3)
FRCS	7 (10)
Other^b^	19 (26)
Designation of podcast hosts	
Trauma surgeon	18 (25)
Surgical resident	1 (1)
Other or not mentioned^c^	53 (74)
**Guests (n = 64)**	
Credentials of podcast guests^a^	
MD	61 (95)
MPH	5 (8)
FRCS	4 (6)
Other^d^	9 (14)
Designation of podcast guests	
Trauma surgeon	46 (72)
Surgical resident	4 (6)
Other^e^	14 (22)

Abbreviations: FRCS, Fellow of the Royal College of Surgeons; MD, Doctor of Medicine; MPH, Master of Public Health.

^a^ Percentages do not equal 100% because each podcast episode included multiple subdomains of clinical knowledge and hosts and guests had multiple credentials.

^b^ Includes medical student, research fellow, Bachelor of Science in Nursing, and Master of Physician Assistant Studies.

^c^ Includes paramedic, registered nurse, nurse practitioner, physician assistant, and digital creator.

^d^ Includes Master of Business Administration, Doctor of Philosophy, Bachelor of Science in Nursing, Doctor of Osteopathic Medicine, and Master of Physician Assistant Studies.

^e^ Includes general surgery physicians, other specialty physician, nurse manager, and physician assistants.

**Figure 2.**
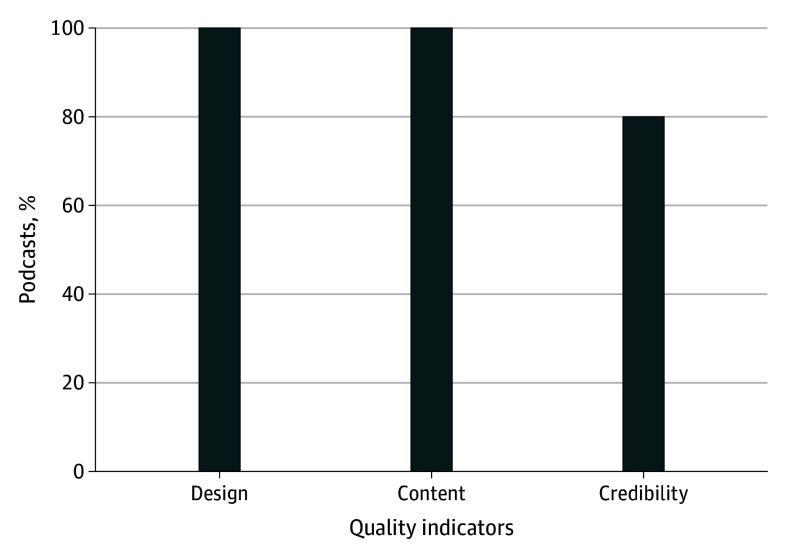
Percentage of Podcasts That Met Quality Domains

Figure 2 shows how well the podcast episodes met the quality indicators. All podcast episodes had excellent quality in terms of content and design. However, for the quality indicators in the credibility domain, 11 of 55 podcast episodes (20%) did not fulfill the credibility criteria.

Table 2 provides a detailed description of quality indicators for all 3 quality indicator domains. Within the credibility domain, 33 of the 55 podcast episodes (60%) did not disclose the conflicts of interest of the hosts and guests; only 22 of the 55 podcasts’ episodes (40%; 95% CI, 30%-50%) fulfilled this quality indicator. Most of the 55 podcast episodes fulfilled other credibility indicators, including disclosing the host’s identity (n = 50 [91%; 95% CI, 80%-100%]), making a distinction between fact and opinion (n = 52 [95%; 95% CI, 80%-100%]) and between advertisement and content (n = 52 [95%; 95% CI, 80%-100%]), and being transparent about the people involved in podcast creation (n = 45 [82%; 95% CI, 70%-90%]). All 55 podcast episodes presented excellent quality content that was accurate, professional, and relevant to the intended audience, with 50 (91%; 95% CI, 80%-100%) podcasts deemed to be an educational resource of good quality. As provided in Table 2, for the design domain, all podcasts used readily accessible technologies to enable access by learners with standard equipment and software.

**Table 2.** Assessment of Podcasts by Specific Quality Indicators

Quality indicator^a^	Podcast episodes (N = 55)	% (95% CI)
Credibility		
Do the authorities (eg, author, editor, publisher) that created the resource list their conflicts of interest?	22	40 (30-50)
Is the identity of the resource’s author clear?	50	91 (80-100)
Does the resource make a clear distinction between fact and opinion?	52	95 (80-100)
Does the resource clearly differentiate between advertisement and content?	52	95 (80-100)
Is the resource transparent about who was involved in its creation?	45	82 (70-90)
Content		
Is the information presented in the resource accurate?	55	100 (90)^b^
Is the content of this educational resource of good quality?	50	91 (80-100)
Is the content of the resource professional?	55	100 (90)^b^
Is the resource useful and relevant for its intended audience?	55	100 (90)^b^
Design		
Does the resource employ technologies that are universally available to allow learners with standard equipment and software access?	55	100 (90)^b^

^a^ Source of quality indicators is Lin et al.^15^

^b^ Since the percentage for podcasts fulfilling the specific indicator is 100%, a 1-sided 95% CI is reported.

## Discussion

In this cross-sectional study that evaluated the quality of trauma surgery podcasts, we found that most of them were developed in high-income countries (HICs) and contributed to clinical knowledge. While most podcast hosts and guests were trauma surgeons with an MD credential, there were considerably fewer female hosts than male hosts. All quality indicators of content and design domains were rated as excellent. However, the credibility domain of the included trauma podcast episodes was not rated up to par, specifically in the context of nondisclosure of conflicts of interest. This finding is congruent with findings reported by Akman et al,^17^ in which most physicians featured in oncology podcasts did not disclose their financial conflicts of interest.

While trauma remains a leading cause of global mortality and disability,^18^ extensive discrepancy is seen within medical students and surgical residents in their exposure to acute care surgery.^19,20^ In instances in which such exposure is required by trainees for educational experience and for pursuing a future subspecialty, podcasts can provide an avenue for gaining knowledge that may not be available otherwise.^21^ Additionally, for surgical trainees with limited time for self-study,^22^ podcasts can provide a convenient method for gaining information and mentorship. However, while surgical trainees globally have limited self-study time and could equally benefit from educational resources such as podcasts, our study found that a substantial number of podcasts are produced in HICs. This finding is consistent with those of other studies,^23,24^ which suggests that the use of medical podcasts is pronounced in HICs compared with low- and middle-income countries (LMICs). While this disparity has not been explored previously for trauma surgery podcasts, financial constraints can potentially limit their production in LMICs.^25^ Furthermore, the difference in trauma presentation, diagnostic modalities, and treatment approaches between HICs and LMICs also limit global use of podcasts.^24^

Our results show that 91% of trauma surgery podcasts provided a clear identity of the host. The quality indicators identified via the modified Delphi consensus indicated that transparency through the host’s identity was one of the most important credibility indicators.^15^ In the present study, 52 of 72 hosts (72%) had an MD degree; these credentials are a vital measure of a host’s capability and the podcast’s quality. A qualitative study from Canada found that listeners of medical podcasts used the hosts’ online profiles to gauge their credibility.^23^ Regarding the sex distribution of hosts within the trauma surgery podcast episodes included in our study, only 15% of hosts were females. A similar pattern has been noted for emergency medicine podcasts published from 2011 to 2021, for which 10.2% (of 3258) hosts were female.^26^ Female physicians have a considerably different experience than their male counterparts globally within the field of surgery.^27,28^ Promoting more female podcast hosts and guests can provide a diverse perspective into surgery, especially for mentorship,^29^ thus increasing the quality of podcast content. While efforts have been initiated in the US to provide podcast mentorship to female clinicians,^29^ similar initiatives need to be taken in low-resource settings to empower female trauma surgeons globally.

We believe that digitized education in the form of podcasts has added immense value to medical learning. However, it is important to determine its legitimacy and quality to ascertain the dissemination of proper information. Standardization of podcast format is also important to ensure the credibility of future podcasts in medicine. Mandating introductions of podcast hosts and guests via specification of their credentials, past experience, and conflicts of interest can increase podcast reliability significantly. The domain of content focused on academic rigor, professionalism, and orientation indicators,^14,15^ all of which were fulfilled by the podcast episodes included in our study. Since credible and accurate information is more important, aesthetics and design play a limited role in establishing the quality of a trauma surgery podcast. Therefore, the design domain assessed only the use of universally available technologies, such as computers for recording and editing and easily accessible software,^30^ which was present in all 55 included podcast episodes.

### Strengths and Limitations

To our knowledge, this is the first study to use systematically identified quality indicators to assess the credibility, content, and design of trauma surgery podcasts. This study has some limitations. While a checklist was used to ascertain the quality of each podcast episode and the data collectors were meticulously trained on its use, the ratings given to each episode still included a subjective element since the domains were independently interpreted and assessed by the residents. Therefore, a senior team member of the research team reviewed the podcast episode to decrease subjectivity within the residents’ responses. Furthermore, this study includes only podcasts on trauma surgery, excluding podcasts within specified subspecialty or trauma focusing on a particular anatomic region. Since podcasts on posttrauma rehabilitation were also excluded, future studies are needed to explore these areas.

## Conclusions

This cross-sectional study found that most trauma surgery podcast episodes have good quality content and design, but many of them did not disclose their conflicts of interest. Medical education podcasts can rapidly provide easy access to clinical knowledge and mentorship to trainees. Such an avenue of digitized education needs to have credible output. Quality assessment of podcasts in medicine can ascertain the dissemination of credible information to listeners. Content creators need to disclose their conflicts of interest appropriately to ensure credibility and improve the quality of their podcasts.

## Notes

Supplement 1. **eTable.** List of Included Podcast Episodes (n = 55)

Supplement 2.
Data Sharing Statement
