# Supplementary material for: Quality of Trauma Surgery Podcasts in Credibility, Content, and Design
Source: JAMA Netw Open. 2024 Jun 20;7(6):e2415636. doi: 10.1001/jamanetworkopen.2024.15636 (PMC11190801; doi:10.1001/jamanetworkopen.2024.15636)
Supplement: Supplement 1. — eTable. List of Included Podcast Episodes (n = 55) [file jamanetwopen-e2415636-s001.pdf]

Supplemental Online Content

Merchant AAH, Shah S, Khursheed AA, et al. Quality of trauma surgery podcasts in credibility, content, and design. *JAMA Netw Open*. 2024;7(6):e2415636. doi:10.1001/jamanetworkopen.2024.15636

eTable. List of Included Podcast Episodes (n=55)

This supplemental material has been provided by the authors to give readers additional information about their work.

eTable. List of Included Podcast Episodes (n=55)

S. No	Podcast Channel	Podcast Episode
1.	Real Life Real Surgery	"Top Knife" miniseries review: Chapter 1 "The 3D Trauma Surgeon" with guest Dr. Michael Mount
2.	Behind The Knife	#127: Mock Orals with Dr. Christian Jones Round 2: Trauma and Acute Care Surgery
3.	Behind The Knife	#153: Trauma, Critical Care & Acute Care Surgery: Behind the Scenes at the "Mattox Conference"
4.	Behind The Knife	#154: Trauma, Critical Care & Acute Care Surgery Day 2
5.	Specialty Stories	28: What is Trauma Surgery? Dr. Darko Shares His Story
6.	Behind The Knife	Big T Trauma Series Ep. 8 - Complex Case Discussion #1
7.	Behind The Knife	Big T Trauma Series Ep. 9 - Complex Case Discussion #2
8.	Behind The Knife	BTK ABSITE #17: Trauma, Part 1
9.	Behind The Knife	BTK ABSITE #18: Trauma, Part 2
10.	The Trauma Podcast	Emergency Medicine & Trauma Surgery: Frenemies for Life - Drs. Scott Weingart & William Teeter
11.	Rocky Mountain Surgery	Episode 15 Teaching on the Trauma Service
12.	Fieldcraft Survival	Episode 313: Kevin Estela is Joined by Dr. Ian Reight, to Discuss his Journey as a Trauma Surgeon
13.	Trauma ICU Rounds	Episode 45 - Modern Insights into an Academic Career in Trauma & Acute Care Surgery with Dr. Carlos Brown
14.	Trauma ICU Rounds	Episode 48 - TRAUMA with Dr. Ken Mattox: Part I
15.	Behind The Knife	Evaluating A Trauma Patient 2/2: Behind The Knife Medical Student and Intern Survival Guide
16.	Behind The Knife	Evaluating a Trauma Patient: Behind The Knife Medical Student and Intern Survival Guide
17.	Trauma Loupes	February 2020
18.	Cold Steel: Canadian Journal of Surgery Podcast	Lawrence Gillman On Coping With Loss
19.	Trauma Loupes	Military Supplement 2020, Episode 100
20.	Journal of Trauma and Acute Care Surgery - Trauma Loupes Podcast	November, Episode 71
21.	Pestana's Surgery Review	Pestana's Surgery Review, Ep. 2 - Trauma
22.	Pestana's Surgery Review	Pestana's Surgery Review, Ep. 3 - Trauma
23.	Pestana's Surgery Review	Pestana's Surgery Review, Ep. 4 - Trauma: Burns and bites
24.	Surgery 101	Trauma - Assessment and Resuscitation
25.	Surgery 101	Trauma - Managing Bleeding
26.	The Dr Coffee Podcast	Trauma Surgery - Coffee with Dr Naadiyah Laher
27.	Critical Care Scenarios	When to Operate in Trauma with Dennis Kim
28.	Geripal Podcast	Zara Cooper the Need to Integrate Geriatrics and Palliative Care into Trauma Surgery
29.	Behind The Knife	BTK ABSITE 2023 - Trauma Part 1 with Dr. Matthew Martin
30.	Behind The Knife	BTK ABSITE 2023 - Trauma Part 2 with Dr. Matthew Martin
31.	Trauma ICU Rounds	Modern Insights into an Academic Career in Trauma & Acute Care Surgery with Dr. Carlos Brown

32.	WarDocs - The Military Medicine Podcast	CAPT Matthew D. Tadlock, MD Surgeons at Sea: Challenges and Innovations - Preparing for Maritime Development
33.	Pragmatic Paramedics	CricEm if you Got Em'
34.	Flatline to lifeline with Dr. Long	Death from Profound Hypothermia and Hemorrhagic Shock
35.	Traumacast The Eastern Association for the Surgery of Trauma	EAST In the Arena - The ACS Full Time Equivalent with Pat Murphy
36.	Traumacast The Eastern Association for the Surgery of Trauma	EAST in the Arena: Metamorphosis Through Burn Care
37.	The Dr. Jeffrey Roth's Looking Good Feeling Great Podcast - Dr Jeffrey Roth & Darrell Craig Harris	Explained - Blunt and Penetrating trauma, plus much more on this new 2023 episode of season 04 on Dr. Jeffrey J. Roth's Looking Good Feeling Great Podcast from Las Vegas.
38.	Trauma ICU Rounds	Frailty, Geriatric Trauma & TBI with Dr. Bellal Jospeh
39.	Emergency Medicine Cases	JJ 22 Laceration Repair - Glue vs Strips vs Staples vs Sutures
40.	Journal of Trauma and Acute Care Surgery - Trauma Loupes Podcast	June 2019 Episode 87
41.	Cold Steel: Canadian Journal of Surgery Podcast	Ken Mattox On Trauma Textbook, TCCACS Conference, And The Courage To Challenge The Status Quo
42.	Journal of Trauma and Acute Care Surgery - Trauma Loupes Podcast	March 2015, Episode 39
43.	Cold Steel: Canadian Journal of Surgery Podcast	Martin Schreiber on Trauma Research and Resuscitation
44.	How It's Med	Med Tech Talks Ep. 74: Dr. Morad Hameed Pt. 1
45.	How It’s Med	Med Tech Talks Ep. 74: Dr. Morad Hameed Pt. 2
46.	Beyond Clean Podcast	Need it Now: Understanding Trauma Surgery & Instrument Needs
47.	Emergency Medical Minute	Podcast 825: ALS vs PD Transport
48.	Orthohub see one do one	Practicing Surgery in South Africa - Sithombo Maqungo
49.	Stay Current in Pediatric Surgery: StayCurrent: Pediatric Surgery	Quick Literature Updates Episode 6
50.	Flatline to lifeline with Dr. Long	Rural Trauma, A Race against Time
51.	Tiger Country: The Trauma Podcast	Subspecialty Trauma & Acute Care Surgeons, or, How Many Fellowships Would A Wood Chuck Do If A Wood Chuck Could Even Be Board-Eligible
52.	Critical Care Scenarios	Trauma resuscitation with Scott Weingart
53.	The Clinical Excellence Podcast	Trauma Surgery
54.	Deep Cuts: Exploring Equity in Surgery	What do surgeon interactions with the police look like in the hospital? - Dr. Priya Prakash, Sharnae Phagan, Dr. William McKinley, Dr. Annie Polcari, and Dr. Lea Hoefer
55.	JAMA Surgery Author Interviews	Whole Blood and Survival in Adults With Severe Hemorrhage at US and Canadian Civilian Trauma Centers
