# Supplementary material for: Quality of Trauma Surgery Podcasts in Credibility, Content, and Design
Source: JAMA Netw Open. 2024 Jun 20;7(6):e2415636. doi: 10.1001/jamanetworkopen.2024.15636 (PMC11190801; doi:10.1001/jamanetworkopen.2024.15636)
Supplement: Supplement 2. — Data Sharing Statement [file jamanetwopen-e2415636-s002.pdf]

Data Sharing Statement

Merchant. Quality of Trauma Surgery Podcasts in Credibility, Content, and Design. *JAMA Netw Open*. Published June 20, 2024. doi:10.1001/jamanetworkopen.2024.15636

Data
Data available: No

Additional Information
Explanation for why data not available: The dataset can be made available on reasonable request.
